# Concentration–QTc Modeling to Support Clinical Development of Fezolinetant

**DOI:** 10.1002/cpdd.1613

**Published:** 2025-10-19

**Authors:** Jace C. Nielsen, Masako Saito, Xuegong Wang, Megumi Iwai, Graeme L. Fraser, Steven Ramael, Jiayin Huang

**Affiliations:** ^1^ Astellas Pharma Global Development, Inc. IL USA; ^2^ Astellas Pharma, Inc. Tokyo Japan; ^3^ Ogeda SA Gosselies Belgium

**Keywords:** concentration–QTc model, fezolinetant, ICH E14, NK3 receptor antagonist

## Abstract

Fezolinetant is a non‐hormonal, selective neurokinin‐3 receptor antagonist that blocks neurokinin B activation of kisspeptin/neurokinin B/dynorphin neurons to thereby modulate neuronal activity in the thermoregulatory center. Fezolinetant has been approved in many regions, including North America, Europe, Asia, and Australia for the treatment of vasomotor symptoms associated with menopause at a dose of 45 mg once daily (QD). The risk of potential QT prolongation for fezolinetant was assessed prior to the initiation of the phase 3 trials. A concentration–QTc (C–QTc) analysis was performed in accordance with recommendations from ICH E14 Guideline and utilized data from a phase 1 single and multiple ascending dose study, which tested single doses up to 900 mg and multiple daily doses up to 720 mg in healthy male and female participants. The fezolinetant C–QTc relationship indicated no clinically relevant QT prolongation at therapeutic or supra‐therapeutic doses of fezolinetant. Based on these modeling results as well as data from other clinical and non‐clinical studies, no thorough QT/QTc (TQT) study was required for fezolinetant.

Vasomotor symptoms (VMS), also known as hot flashes or night sweats, involve sudden sensations of heat in the upper body, particularly in the head, neck, chest, and upper back. The thermoregulatory center in the hypothalamus is innervated by kisspeptin/neurokinin B/dynorphin (KNDy) neurons, the activity of which is modulated in a balanced manner by estrogen‐mediated inhibition and neuropeptide neurokinin B (NKB)‐mediated stimulation. During menopause, declining estrogen levels lead to an imbalance, resulting in overstimulation of KNDy neurons, and subsequent activation of peripheral warm sensing neurons.^1^

Fezolinetant is a non‐hormonal, selective neurokinin‐3 (NK3) receptor antagonist that blocks NKB activation of KNDy neurons to thereby modulate neuronal activity in the thermoregulatory center and reduce VMS.^2^ Results from a phase 2b clinical study showed substantial, dose‐dependent reductions in the frequency and severity of VMS for fezolinetant compared to placebo.^3^ Subsequent phase 3, placebo‐controlled studies, SKYLIGHT 1 (NCT04003155),^4^ SKYLIGHT 2 (NCT04003142)^5^ and long‐term safety study, SKYLIGHT 4 (NCT04003389)^6^ confirmed the clinical efficacy and safety of fezolinetant and supported the approval of fezolinetant in many regions, including North America,^7^, ^8^ Europe,^9^, ^10^ Asia,^11^ and Australia^12^ for the treatment of VMS associated with menopause at a dose of 45 mg once daily (QD).

Prior to initiation of the phase 2 and phase 3 programs for fezolinetant, single and multiple ascending dose studies were conducted to assess the pharmacokinetics, safety, and maximum tolerated dose for fezolinetant in healthy men and women. Both men and women were included due to the potential for indications in sex hormone disorders for both sexes. These studies included single doses of up to 900 mg and multiple doses of up to 720 mg.

Fezolinetant is absorbed rapidly with maximum concentrations occurring between 1 and 4 h (median 1.5 h) after dose administration.^7^ Fezolinetant is primarily metabolized by CYP1A2 and, to a lesser extent, by CYP2C9 and CYP2C19.^7^ The major metabolite, ES259564, is the only metabolite identified in human plasma and is approximately 20‐fold less potent against the human NK3 receptor and has no significant off‐target activity (data on file). The effective half‐life of fezolinetant in women with VMS is 9.6 h. Fezolinetant and ES259564 plasma concentrations increase in a proportional manner with dose in the therapeutic dose range. The majority of the fezolinetant dose is excreted in the urine as ES259564.^7^

Cardiovascular safety and potential QT interval prolongation was evaluated as part of the assessment for safety in the phase 1 data. The ICH E14 guidance and subsequent Question and Answers (Q&As) Revisions^13^, ^14^ have highlighted the opportunity for concentration–QTc (C–QTc) data from phase 1 dose escalation studies to be used as an alternative approach for characterizing the QT prolongation risk of a new drug candidate. Hence, we assessed the relationship between fezolinetant and its major metabolite plasma concentrations and change from baseline Fridericia‐corrected QT (dQTcF) intervals to assess QT liability in lieu of a thorough QT/QTc (TQT) clinical study. In this report, we describe the C–QTc modeling results for an early phase 1 study investigating supra‐therapeutic doses of fezolinetant.

## Methods

### Clinical Study Design

A phase 1, single and multiple ascending dose study of fezolinetant (Study ESN364‐CPK‐102, EudraCT Number 2015‐005739‐42) was conducted at a clinical site of SGS life sciences (Antwerp, Belgium) from February 2016 to July 2016. This study was conducted in accordance with the principles of the Declaration of Helsinki, Good Clinical Practice, and International Council for Harmonization of Technical Requirements for Pharmaceuticals for Human Use guidelines. The protocols, its amendments, and informed consent forms were reviewed and approved by the institutional review board. All participants provided written informed consent for participation in these studies.

This study was a single‐center, double‐blind, placebo‐controlled, single‐ and multiple‐dose escalation study of fezolinetant in healthy male and female volunteers consisting of three parts. Healthy volunteers with no clinically significant abnormalities as determined by medical history, physical examination, blood chemistry assessments, hematologic assessments, coagulation and urinalysis, measurement of vital signs, and ECGs were enrolled in this study.

#### Part 1

Healthy pre‐ and postmenopausal female participants in Part 1 received single ascending doses of fezolinetant. A total of 16 female participants (Panels A and B, each consisting of 8 female participants), were randomized to receive either fezolinetant or matching placebo in a 6:2 ratio at each dose level. Each panel was to include single doses of fezolinetant under fasting conditions in up to four treatment periods following an alternating panel design. During each period, participants were residential at the clinical unit from the day before dosing until 48 h after study drug intake. A 6‐day washout was enforced between treatment periods. The last study period was followed by a post study visit 7 to 10 days after the last dose in the last study period. Meals were standardized on the day of dosing across the different treatment periods. The doses of fezolinetant that were tested included 180, 540, and 900 mg for Panel A and 360, 720, and 900 mg for Panel B.

Note that based on review of the pharmacokinetic and safety data, the 900 mg fezolinetant dose tested in Panel A was regarded as the MTD. Therefore, the participants in Panel B also received a single 900 mg dose of fezolinetant in their third period.

#### Part 2

Healthy female participants received multiple ascending doses of fezolinetant in Part 2 of the study. A total of 16 female participants (Panels C and D, each consisting of 8 female participants) were administered fezolinetant or matching placebo in a 6:2 ratio, QD for 7 days with a follow‐up visit 7 to 10 days after the last dose. Participants received fezolinetant under fasting conditions on Day 1 and Day 7 and under fed conditions for the other days in the study. The dose levels evaluated in Part 2 included 540 and 720 mg QD.

#### Part 3

Healthy male participants received single oral doses of fezolinetant in Part 3. Eight participants in one panel (Panel E) were randomized to receive either fezolinetant or matching placebo in a 6:2 ratio under fasted meal conditions. The doses evaluated in this single dose panel included 720 and 900 mg.

### ECG Measurements

Triplicate 12‐lead ECGs were recorded after 5 min in a supine position with approximately 1‐min intervals between the recordings. In Part 1 and Part 3, triplicate ECGs were performed at pre‐dose, 1, 2, 4, 8, and 24 h post‐dose on the days of study drug administration. In Part 2, triplicate ECGs were performed at pre‐dose, 1, 2, 4, 8, and 24 h post‐dose on Day 1 and Day 7 as well as 2 h post‐dose on Day 3 and Day 5. The ECGs were recorded from a single lead (primarily lead II) and were provided to a central laboratory (Clario [formerly ERT Clinical], Peterborough, UK) as aggregate beat data. The paper speed was 25 mm/s, so that the different ECG intervals could be measured manually. XML reports of ECGs were collected and saved. The initial interpretations of the ECGs for safety assessments were performed by the investigator or designee at the study center. ECGs used in C–QTc analysis were manually adjudicated by a central laboratory.

### Pharmacokinetics

Blood samples of approximately 2 mL were collected at pre‐dose, 1, 1.5, 2, 3, 4, 6, 8, 12, 16, 24, and 48 h after dosing on Day 1. For the multiple dose cohorts in Part 2, an additional pre‐dose sample on Day 5 as well as pre‐dose, 1, 1.5, 2, 3, 4, 6, 8, 12, 16, 24, and 48 h after dosing on Day 7 were collected. Immediately after blood collection, the plasma was separated in a refrigerated centrifuge (4°C–8°C) for 10 min at 1500 g and stored below −20°C.

Plasma concentrations of fezolinetant were measured using a high‐performance liquid chromatography‐tandem mass spectrometry assay (LC‐MS/MS) in SGS Life Science (Wavre, Belgium). The method was validated over a range of 5.00 to 4000 ng/mL for fezolinetant, and the lower limit of quantification (LLOQ) was 5.00 ng/mL. Plasma concentrations for ES259564 were analyzed using leftover samples only for Parts 1 and 2 by a non‐validated assay method.

### Concentration–QT Modeling

The relationship between time‐matched fezolinetant and ES259564 plasma concentrations and change from baseline QT intervals was evaluated using a predefined linear mixed effect model described in a white paper by Garnett et al.^15^ QT intervals were corrected for heart rate using Fridericia's correction (QTcF). Triplicate QTcF intervals were averaged and rounded to the nearest integer prior to the C–QTc analysis. Participants who received placebo were included in the analysis and assigned a zero value for plasma concentrations. Plasma concentrations that were below the limit of quantification were included in the analysis and set to zero. Only data with matching concentration and ECG values were used in the analysis; missing matched data were not included.

#### Exploratory Assessments

Prior to model‐based analysis, the following graphical examination was performed to assess key assumptions about the relationship between fezolinetant and ES259564 plasma concentrations and QTc interval data.

1. The lack of drug effects on heart rate were examined by plotting mean change from baseline over time stratified by day and treatment group.
2. The suitability of the Fridericia method for heart rate corrected QT (QTc) intervals was evaluated by a scatter plot of QTcF intervals versus RR intervals. If plots suggested adequate correction using Fridericia method, no further evaluation of alternative methods was undertaken.
3. Potential temporal delays between drug concentration and effects were identified by graphical examination of hysteresis plots. In addition, the mean fezolinetant and ES259564 plasma concentration versus nominal time profile was compared to mean changes in dQTcF over nominal time.
4. The assumption of a linear relationship between concentration and effect was tested using a scatter plot of dQTcF versus drug and metabolite concentration overlaid with a trend line using a nonparametric LOESS smoother.

#### Modeling Approach

A predefined linear mixed effect model was used to characterize the relationship between drug concentrations and dQTcF intervals.^15^ The fixed effects in the model included: intercept, treatment, day, nominal time, deviation of baseline from the overall mean baseline, and drug concentration. An interaction term was also estimated between day (i.e., Day 7) and drug concentration slope. The inclusion of the interaction parameter on concentration slope was pre‐specified due to the multiple dose design in Part 2 and aimed to account for potential changes in drug effects over time. Variables treated as class effects were: day (0 = Day 1, 1 = Day 7), nominal time (0, 1, 2, 4, 8, and 24) and treatment (placebo and fezolinetant). Subjects were treated as random effects. Baseline was defined as the average of the triplicate ECG measured prior to treatment administration on Day 1 in each period. Participants in Part 1 and Part 3 typically participated in multiple periods, as such, the average baseline for each individual was calculated prior to calculating the grand mean baseline from all participants. An unstructured variance–covariance matrix was used for the random effects on intercept and concentration slope. Model parameters were estimated using maximum likelihood. The numerator degrees of freedom were estimated using Kenward–Rogers approximation. In this analysis, the overparameterized model was only simplified if the model did not converge. This approach was pre‐specified and undertaken to avoid unnecessary model building steps in situations when the inflated confidence intervals for the overfit model do not indicate a QT liability.

Two different models were considered including one model that evaluated only fezolinetant concentrations and a separate model that evaluated the effect of fezolinetant and of the primary metabolite, ES259564. Two different models were evaluated because ES259564 plasma concentrations were not available for Part 3 of the study and because ES259564 pharmacokinetic concentrations were not analyzed using a validated bioanalytical assay. Available ES259564 concentrations were assessed in order to fully explore the impact of fezolinetant administration on potential QTcF prolongation.

The first model, referred to as the Parent Model, included only the effect of fezolinetant plasma concentration on dQTcF. The second model, referred to as the Parent and Metabolite Model, included fixed effects for both fezolinetant and ES259564. Note that since ES259564 concentration data was not available for Part 3, only Part 1 and Part 2 are included in the Parent and Metabolite Model. Equations for the Parent Model and Parent and Metabolite Model are provided in Equations (1) and (2), respectively.

(1)

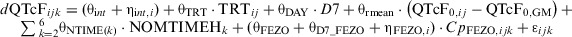



(2)

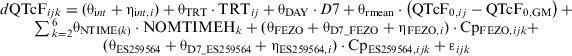

where *θ*_int_ is the intercept (ms), *θ*_TRT_ is the treatment effect (ms), *θ*_DAY_ is the change in intercept for Day 7 (ms), *θ*_rmean_ is the effect of regression to the mean, *θ*_NTIME(_ *_k_*_)_ is the parameter estimate associated with the *k*th nominal time (NOMTIMEH), *θ*_Fezolinetant_ is the fezolinetant slope (ms/(µg/mL)), *θ*_D7_Fezolinetant_ is the change in fezolinetant slope for Day 7 (ms/(µg/mL)), *θ*_ES259564_ is the ES259564 slope (ms/(µg/mL)), *θ*_D7_ES259564_ is the change in ES259564 slope for Day 7 (ms/(µg/mL)), TRT*_ij_* is the indicator variable for treatment (Placebo = 0, Fezolinetant = 1) in *i*th participant at *j*th period, D7 is the indicator variable for Day 7 (Day 1 = 0, Day 7 = 1), CP_FEZO,_ *_ijk_* is the fezolinetant plasma concentration (µg/mL) in *i*th participant at *j*th period at *k*th time point, CP_ES259564,_ *_ijk_* is the ES259564 plasma concentration (µg/mL) in *i*th participant at *j*th period at *k*th time point, QTcF_0,_ *_ij_* is the individual baseline QTcF in *i*th participant at *j*th period, QTcF_0,GM_ is the grand mean baseline QTcF interval for all participants, *η*_int_ is the participant‐specific random effect on intercept, *η*_Fezolinetant_ is the participant‐specific random effect on fezolinetant slope, *η*_ES259564_ is the participant‐specific random effect on ES259564 slope, and *ε_ijk_* is the residual error.

The ability of the model to describe the QTcF interval data was assessed based on a standard battery of goodness‐of‐fit diagnostics, the stability of the model, ability of the model to converge, and precision of model parameter estimates.

#### Model‐Based Prediction of ddQTc

Predictions of the magnitude of ddQTcF and potential QT liability were made at clinical concentrations of interest. These include the therapeutic concentration, the highest anticipated clinical exposure based on covariate effects, formulation differences, and specific populations (renal and hepatic impairment), which is 6 times the therapeutic concentration, and 10 times the therapeutic concentration. A 6‐fold increase in C_max_ was determined to be the supra‐therapeutic exposure based on known and unknown covariates anticipated to increase fezolinetant or ES259564 exposure. Known covariate effects included the tablet formulation, participants receiving weak CYP1A2 inhibitors, Asian participants, and participants with lower body weight (e.g., 45 kg). Note that strong and moderate CYP1A2 inhibitors were to be contraindicated for concomitant use with fezolinetant and were therefore not included in the clinical exposure predictions. Combined, these covariate effects accounted for an approximately 3‐fold increase in fezolinetant C_max_. In addition, to account for the unknown effect of renal and hepatic impairments at the timing of the analysis, a 1.82‐fold multiplier was further included based on the observed interaction of strong CYP1A2 inhibitor. Based on the same rationale and data observed for the effects of CYP1A2 inhibitors and smoking on the pharmacokinetics of ES259564, a 6‐fold increase in C_max_ was also considered sufficient to cover the supra‐therapeutic concentration of ES259564. Finally, note that the 10‐fold increase was included as a sensitivity analysis to further ensure that any additional increase in fezolinetant or ES259564 plasma concentration had no impact on the QT interval. The predicted mean and its upper one‐sided 95% CI for ddQTcF were calculated using the ESTIMATE statement in the SAS MIXED procedure.

#### Software

Data set preparation, modeling development predictions, and postprocessing of model output were conducted using SAS for Windows v.9.4 (SAS Institute, Inc., Cary, NC). Plots were created using R (Version 3.4.4, R Foundation for Statistical Computing, Vienna, Austria).

## Results

### Demographics

A total of 40 participants including 32 healthy female participants and 8 male participants were enrolled in this study and included in the analyses. Participants were predominately white with ages ranging from 22 to 53 years (Table 1). All 40 randomized and treated participants completed the study.

**Table 1:** Summary of Demographics

	Part 1		Part 2		Part 3	
Demographics	Panel A (N = 8)	Panel B (N = 8)	Panel C (N = 8)	Panel D (N = 8)	Panel E (N = 8)	Total
**Age, years** Median (min, max)	42.0 (22–53)	29.5 (23–53)	37.5 (30–50)	36.5 (25–50)	33.5 (28–41)	34.5 (22–53)
**Height, cm** Median (min, max)	169.1 (158–178)	166.3 (159–172)	166.7 (158–176)	168.7 (160–175)	182.8 (171–194)	168.9 (158–194)
**Weight, kg** Median (min, max)	62.15 (59.0–76.2)	66.30 (56.2–81.4)	63.95 (55.3–71.6)	74.95 (65.5–88.2)	75.55 (63.2–94.6)	68.75 (55.3–94.6)
**BMI, kg/m^2^** Median (min, max)	22.65 (20.7–26.2)	23.55 (20.2–28.8)	22.80 (20.6–27.1)	26.45 (23.1–29.0)	22.85 (19.7–27.8)	23.20 (19.7–29.0)
**Sex,** N (%) Female Male	8 (100) 0 (0.00)	8 (100) 0 (0.00)	8 (100) 0 (0.00)	8 (100) 0 (0.00)	0 (0.00) 8 (100)	32 (80.0) 8 (20.0)
**Race,** N (%) Black or African American White	0 8 (100)	0 8 (100)	1 (12.5) 7 (87.5)	1 (12.5) 7 (87.5)	0 8 (100)	2 (5.0) 38 (95.0)

### Safety and Tolerability

No serious treatment‐emergent adverse events (TEAEs) or deaths occurred during this study and no TEAEs for which the study drug was temporarily or permanently stopped were reported.

No treatment‐emergent QTcF values of more than 450 ms were observed in any of the three study parts. One (16.7%) participant in Part 1 experienced an increase in QTcF interval of 32 ms compared to baseline levels following administration of 540 mg fezolinetant on Day 1, 4 h post‐dose. This increase of ≥30 ms was only observed once, and the participant completed the study. No other participants had a QTcF increase of ≥30 ms. None of the abnormalities were considered as clinically significant. None of the abnormalities were reported as AE.

### Pharmacokinetics

**Figure 1:**
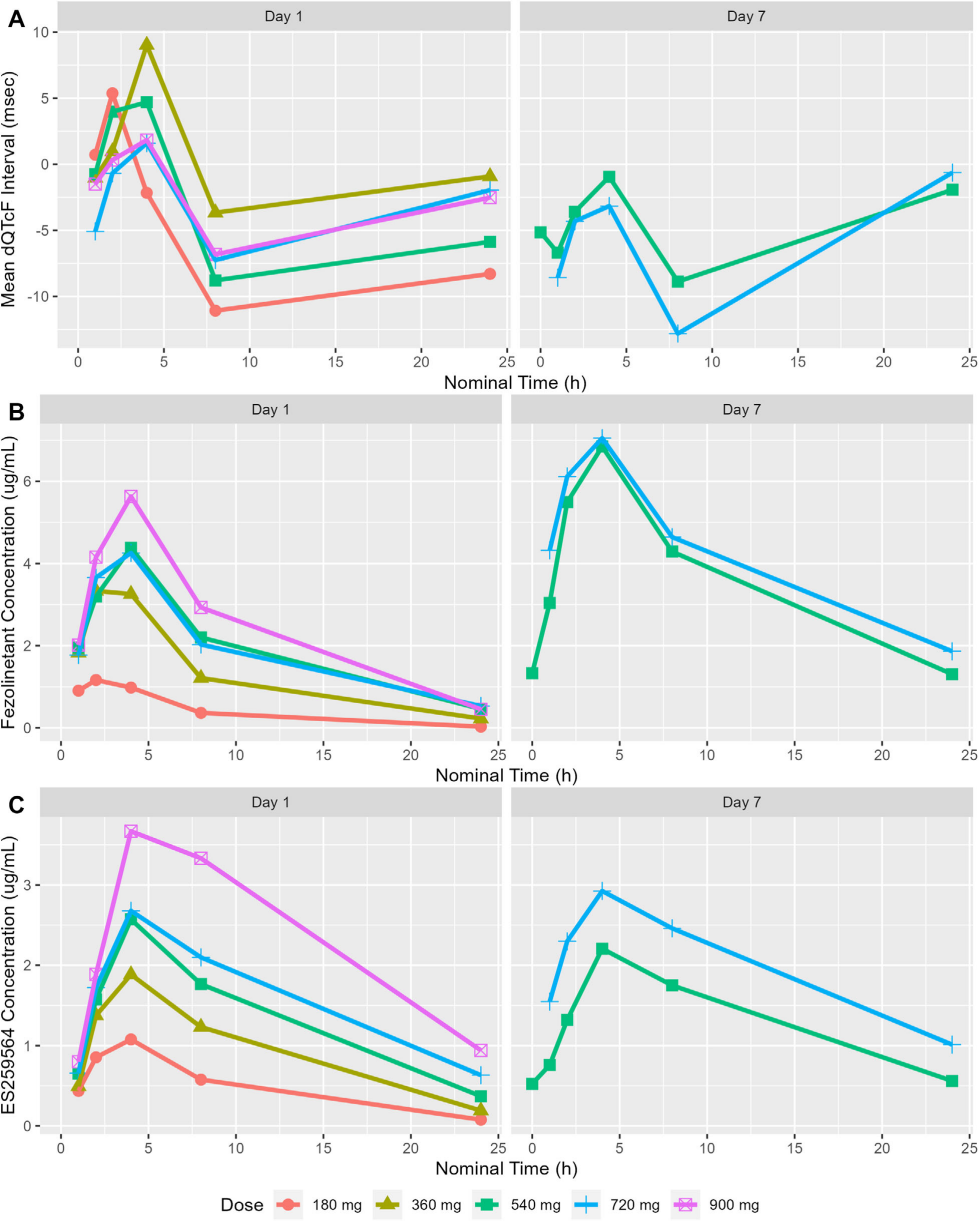
Mean change from baseline QTcF, plasma fezolinetant, and ES259564 concentration–time profiles on Day 1 and Day 7. Top panels (A) are mean dQTcF Interval (ms), middle panels (B) are the mean fezolinetant plasma concentrations, and bottom panels (C) are the mean ES259564 plasma concentrations over time grouped by dose on Day 1 and Day 7.

Mean fezolinetant and ES259564 maximum plasma concentrations were reached after 3 to 4 h. Mean concentration–time profiles showed a biphasic elimination for all dose groups (Figure 1).

### Concentration–QT Interval Modeling

The primary analysis (Parent Model) evaluating the impact of fezolinetant plasma concentrations on QTcF included 456 observations from 40 participants in Parts 1, 2, and 3 of Study ESN364‐CPK‐102. The secondary analysis (Parent and Metabolite Model) including both fezolinetant and ES259564 plasma concentrations included 377 observations from 32 participants that participated in Part 1 and Part 2. Data from Part 3 were excluded because ES259564 concentrations were not available.

#### Exploratory Assessments

**Figure 2:**
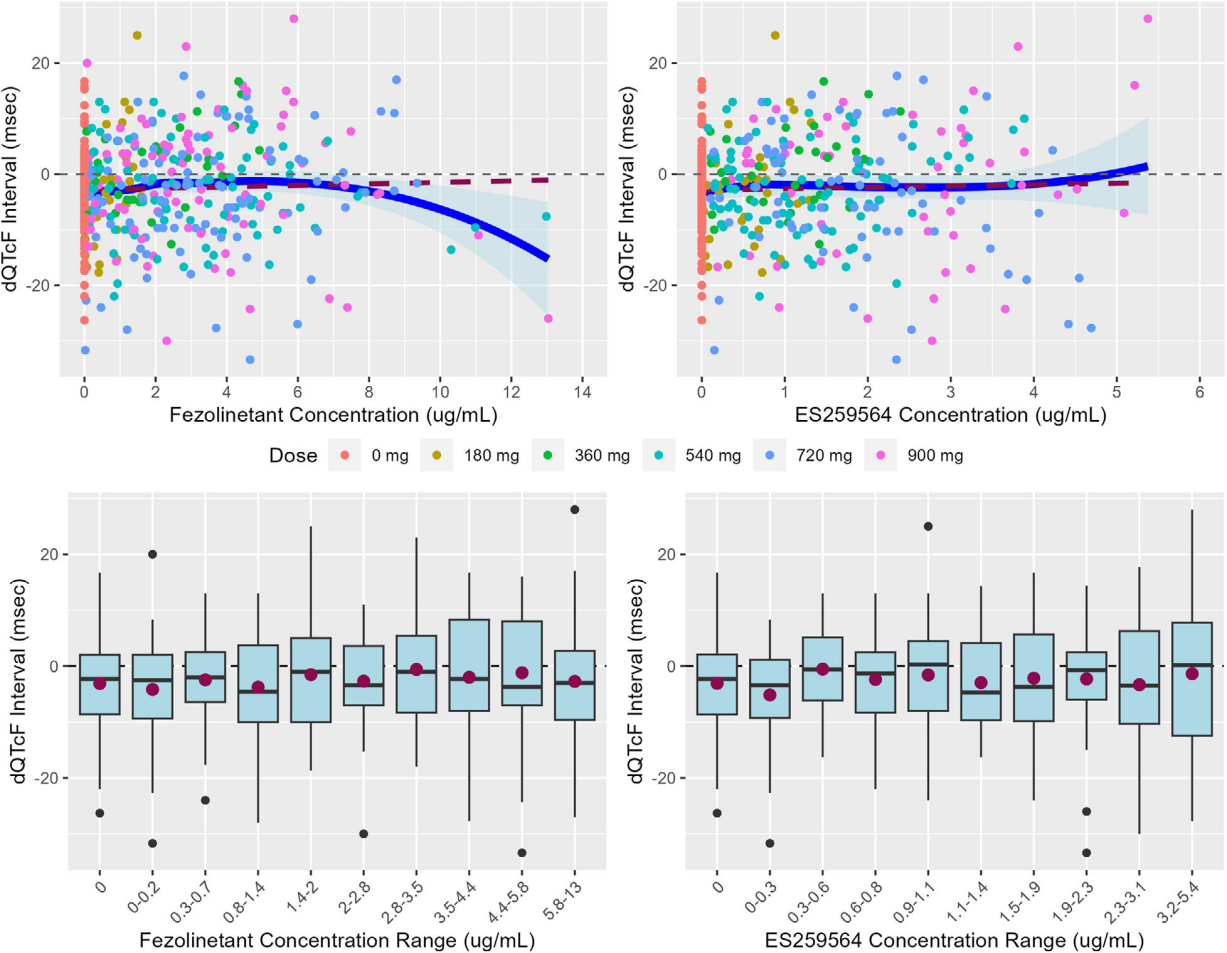
Linearity of PK/PD relationship.

Key assumptions for the pre‐specified linear mixed effect model were evaluated using exploratory plots. The time course of mean change from baseline in heart rate by dose showed no meaningful difference between groups (Figure S1). No trend was observed between QTcF intervals and RR intervals indicating the correction factor was adequate to control for heart rate (Figure S2). The hysteresis plots give no clear indication of a temporal delay between concentration and effect (Figure S3). In addition, the maximum change in mean dQTcF over time coincided with the maximum plasma concentration for both fezolinetant and ES259564 (Figure 1). Finally, a linear relationship was observed in a scatter plot of dQTcF versus fezolinetant and ES259564 (Figure 2). These plots supported the application of the pre‐specified linear mixed effect model.

#### Linear Effect Model

The predefined Parent Model described in Equation (1) converged. The model included all the previously described fixed effects as well as an unstructured variance–covariance matrix for the random effects. The fixed effects for treatment, deviation from baseline, and nominal sample times 2, 4, and 8 were identified as statistically significant parameters. The fezolinetant concentration slope was not statistically significant and was estimated to be negative. The point estimate (95% CI) for the fezolinetant concentration slope was −0.5071 (−1.1279, 0.1137) ms/(µg/mL) on Day 1 and −0.3622 (−1.0851, 0.3607) ms/(µg/mL) on Day 7. Parameter estimates for the Parent Model are listed in Table S1.

The predefined Parent and Metabolite Model described in Equation (2) did not converge. The variance–covariance matrix for the random effects was subsequently simplified to a diagonal structure. The revised model including all the predefined fixed effects converged. The slope of the concentration change from baseline QTcF interval relationship was statistically significant (*P* < .05) for both the parent and metabolite on Day 1, but not Day 7. The slope for fezolinetant was negative and the point estimate (95% CI) was −1.5319 (2.3226, −0.7411) ms/(µg/mL) and −0.7255 (−1.5660, 0.1151) ms/(µg/mL) for Day 1 and Day 7, respectively. The slope for ES259564 was positive and the point estimate (95% CI) was 2.4566 (1.0202, 3.8930) ms/(µg/mL) and 0.7740 (−1.5010, 3.0490) ms/(µg/mL) for Day 1 and Day 7, respectively. Other statistically significant fixed effects parameters (*P* < .05) included deviation from baseline, and nominal time 2, 4, and 8. Parameter estimates for the Parent and Metabolite Model are listed in Table S2.

**Figure 3:**
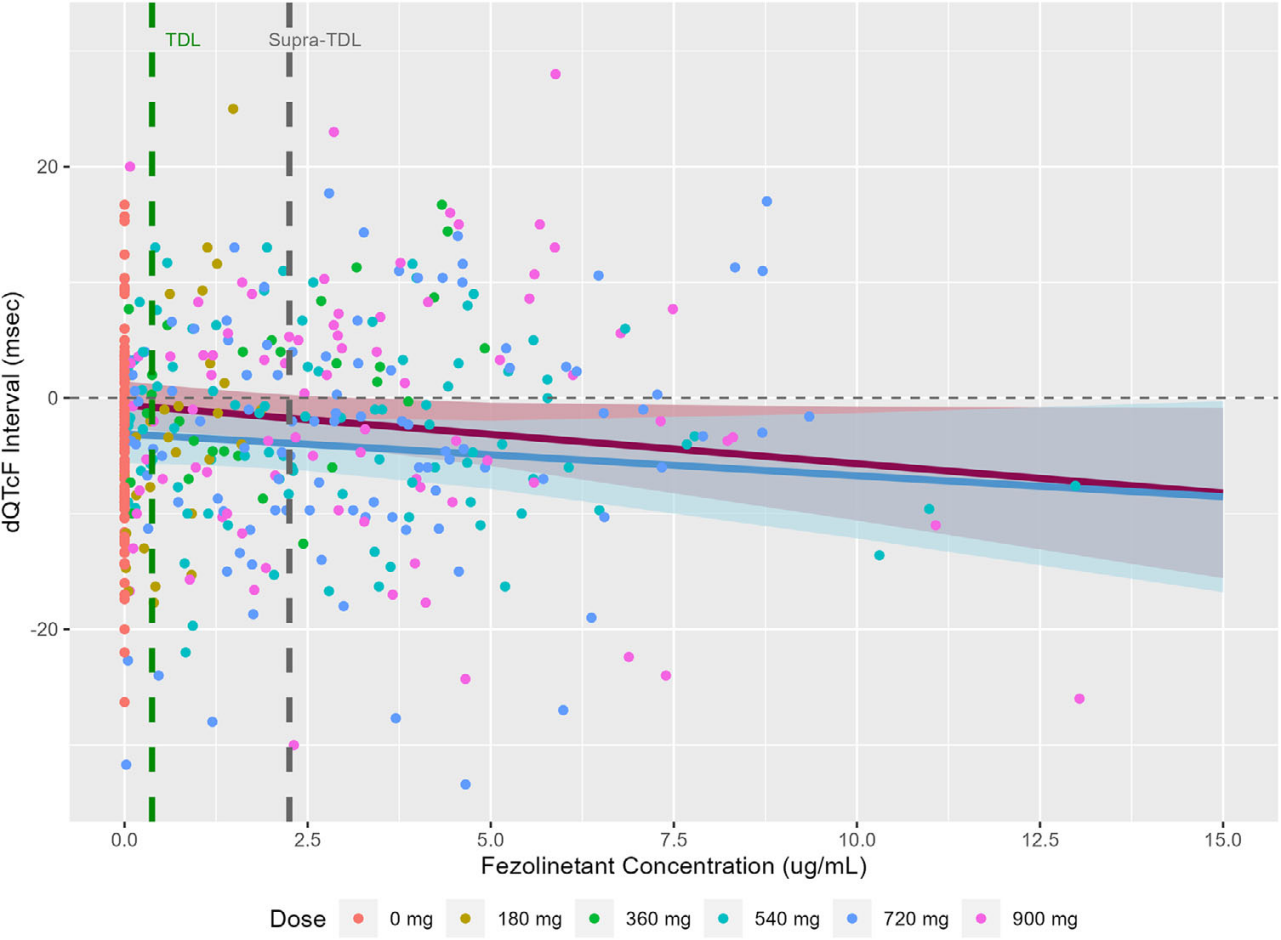
Concentration‐QTcF plots for the Parent Model. Solid red line (pink shaded region) is the population mean model prediction (90% CI) for Day 1. Solid blue line (blue shaded region) is the population mean model prediction (90% CI) for Day 7. Colored symbols are the observed dQTcF intervals. Vertical dashed green line represents the concentration for the therapeutic dose level (TDL) and the vertical dashed grey line represents the concentration for the supra‐therapeutic dose level.

**Figure 4:**
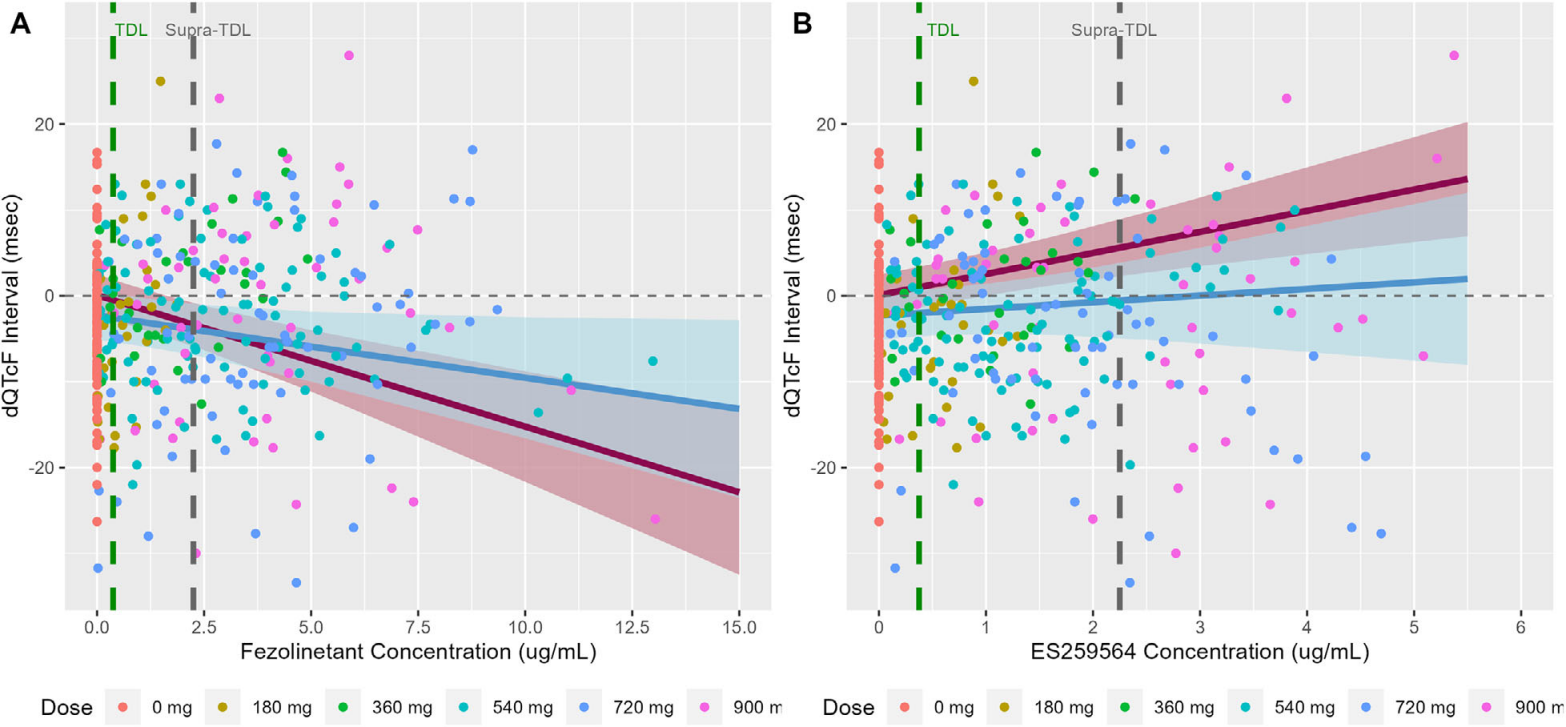
Concentration–QTcF plots for the Parent and Metabolite Model. Solid red line (pink shaded region) is the population mean model prediction (90% CI) for Day 1. Solid blue line (blue shaded region) is the population mean model prediction (90% CI) for Day 7. Colored symbols are the observed dQTcF intervals. Vertical dashed green line represents the concentration for the therapeutic dose level (TDL) and the vertical dashed grey line represents the concentration for the supra‐therapeutic dose level.

Parameter estimates from the Parent Model and the Parent and Metabolite Model were used to generate predictions to illustrate the effect of fezolinetant and ES259564 concentration on the dQTcF and ddQTcF. Figure 3 shows the population mean prediction and 90% CI for dQTcF versus fezolinetant concentration on Day 1 and Day 7 for the Parent Model. The observed dQTcF measurements are overlaid. There was considerable overlap in the predictions for Day 1 and Day 7 indicating a lack of time effect for the parent concentration slope. Similar plots were also prepared for the Parent and Metabolite Model. The separate effects of fezolinetant and ES259564 concentration on dQTcF interval are presented in Figure 4. The predicted effects of fezolinetant concentration on dQTcF in the Parent and Metabolite Model were similar to those observed in the Parent Model, but with a slightly more negative slope for both Day 1 and Day 7. The predicted independent effects of ES259564 on dQTcF show a positive slope on both Day 1 and Day 7. A larger effect was noted on Day 1 compared to Day 7; however, there was still some overlap in the CIs. The negative slope for fezolinetant concentration and the positive slope for ES259564 concentration suggests a relatively neutral combined effect at equal concentrations of parent and metabolite in the anticipated therapeutic and highest clinical exposure range following fezolinetant administration.

#### Model Diagnostics

Diagnostic plots for the Parent Model (Figure S4) and the Parent and Metabolite Model (Figure S5) showed that residuals were generally evenly distributed around the zero line and approximately normally distributed. No trends in residuals were evident for nominal time point or treatment group. A comparison of the observed and individual predicted dQTcF values is also provided and suggests good agreement between observed and individual predicted values. Overall, these results indicate that the predefined model adequately describes the observed dQTcF data.

#### Prediction of Model‐Based ddQTc

The model predicted ddQTcF values and upper one‐sided 95% CIs for the anticipated therapeutic concentration, highest clinical exposure, and 10 times the therapeutic concentration are provided for the Parent Model and the Parent and Metabolite Model in Table 2. The therapeutic concentration (approximately 0.375 µg/mL) represents the anticipated steady‐state mean C_max_ for fezolinetant and ES259564 from a 45 mg QD dose of fezolinetant. These values were derived based on the observed mean C_max_ (0.244 µg/mL for fezolinetant and 0.246 µg/mL for ES259564 at 30 mg QD) in a DDI study^16^ and scaled assuming linear pharmacokinetics. The highest clinical exposure (i.e., 6 times the therapeutic concentration for both fezolinetant and ES259564) represents the highest anticipated clinical exposure (supra‐therapeutic exposure) considering covariate effects (use of CYP1A2 weak inhibitors, lower body weight, and Asian race), formulation effects (capsule used in early phase 1 studies vs tablet), and disease effects to account for special population (e.g., hepatic and renal impairment) for both fezolinetant and ES259564. The upper one‐sided 95% CI did not exceed 10 ms at 10 times the therapeutic plasma concentrations for predictions from either the Parent Model or the Parent and Metabolite Model.

**Table 2:** Summary of ddQTcF Predictions for Relevant Concentrations From the Parent Model and the Parent and Metabolite Model

		Parent Model		Parent and Metabolite Model	
	Day	Prediction (ms)	Upper One‐Sided 95% CI (ms)	Prediction (ms)	Upper One‐Sided 95% CI (ms)
Therapeutic concentration (0.375 µg/mL)	1	2.0794	0.3276	1.4536	3.3483
	7	2.1337	0.3421	1.1251	3.1085
Highest clinical exposure (6‐times therapeutic concentration) (2.25 µg/mL)	1	1.1287	−0.546	3.1875	5.4323
	7	1.4546	−0.5574	1.2162	4.8564
10‐Times therapeutic concentration (3.75 µg/mL)	1	0.3681	−1.6262	4.5745	7.8686
	7	0.9114	−1.652	1.2890	7.0062

## Discussion

The phase 1, single and multiple ascending dose study described in this report was designed to assess the pharmacokinetics, safety, and tolerability of fezolinetant in healthy men and women but was also suitable for evaluation of the relationship between fezolinetant and primary metabolite exposure and changes in the QT interval because of several key design features. First, the wide dose range studied helped to ensure adequate characterization of the exposure response relationship. Second, the large multiple between the highest dose evaluated (i.e., 900 mg) and the therapeutic dose used in phase 3 clinical trials (i.e., 45 mg) obviated the need for a separate positive control because the observed pharmacokinetic concentrations for both the parent and metabolite maintained a sufficient exposure margin by exceeding the highest expected clinical exposure by more than 2‐fold.^15^ Finally, ECGs were collected and read with sufficient quantity and quality to support such a quantitative assessment.

The relationship between change from baseline QTcF and fezolinetant and ES259564 plasma concentrations was assessed in humans using linear mixed effects models. The pre‐specified models adequately characterized the data including differences in study control procedures (placebo effect, circadian changes, and food effects). Combined parent and metabolite models were investigated because no in vitro data was available with regard to potential QT prolongation for the major metabolite. Simulation studies have shown that accurate parameter estimates can be achieved in a joint model even when parent and metabolite concentrations are correlated.^17^ The correlation between parent and metabolite concentrations in this analysis was 0.65 and well below what would be considered problematic for parameter estimation in the context of collinearity. Univariate models including parent and metabolite separately were not considered since simulation studies have showed this approach can lead to biased parameter estimates for both analytes in some situations.^17^ As shown in Figures 3 and 4, the fezolinetant concentration slope was negative while the ES259564 concentration slope was positive. In addition, changes in the parent and metabolite slope based on single dosing versus multiple dosing were predicted. While these effects were pre‐specified and included in the model to eliminate potential bias in slope parameters over time, the differences in slope were relatively small in the context of the uncertainty of the predictions. Importantly, model predictions of the upper one‐sided 95% CI for ddQTcF did not exceed 10 ms for the therapeutic concentration, highest clinical exposure, or the 10‐times therapeutic concentration for either model. These results suggest that fezolinetant has no clinically meaningful effect on QT interval prolongation in humans at Day 1 or at Day 7.

The findings from C–QTc modeling are consistent with results from non‐clinical studies for fezolinetant. Non‐clinical studies on the cardiovascular system were performed using HEK293 cells transfected with human ether‐a‐go‐go‐related gene (hERG), conscious cynomolgus monkeys implanted with a telemetry system, and isolated rabbit hearts (Langendorff study). In the hERG assay, fezolinetant inhibited the potassium current in a dose related manner, however, the IC_50_ value was 371‐fold higher the human therapeutic exposure (C_max,u_). In the telemetry study in cynomolgus monkeys, there were no significant effects on any parameters up to 59‐fold for fezolinetant and 29‐fold for ES259564 compared with the human therapeutic PK exposure. In addition, the Langendorff study revealed that fezolinetant did not affect any electrophysiological or mechanical parameters at concentrations up to 48‐fold the human therapeutic exposure. In repeated dose toxicity studies in cynomolgus monkeys, there were no effects on electrocardiography or blood pressure for 39 weeks. Note that as the plasma protein binding ratio of fezolinetant was lower in animals, these comparisons were made using total exposure. Overall, non‐clinical studies did not suggest a cardiac risk at the anticipated clinical exposure for fezolinetant.^18^

The C–QTc analysis findings were submitted to regulators, including the FDA's Interdisciplinary Review Team, as part of a broader package which included additional clinical and non‐clinical data at the end of phase 2. The totality of this data supported a waiver of a dedicated thorough QT study and enabled the subsequently conducted phase 3 clinical trials to be designed with standard cardiovascular and ECG monitoring. No clinically relevant changes in the quantitative interval measures of ECG parameters were observed in these pivotal studies.^19^ In clinical studies to date, none of the events identified to be of clinical importance per the ICH E14 guidelines (i.e., syncope, seizure, significant ventricular arrhythmias, or sudden cardiac death) occurred on fezolinetant 45 mg QD treatment.

## Conclusions

Fezolinetant was safe and well tolerated in healthy volunteers up to 900 mg. The relationship between fezolinetant and/or ES259564 concentration and dQTcF was evaluated using data spanning a wide dose range with digital triplicate ECG recordings and manually adjudicated readings. The predictions from the predefined linear mixed effect model suggested no clinically relevant ddQTcF at the 10 times of maximum concentrations for the therapeutic dose of 45 mg. Subsequent pivotal phase 3 studies confirmed the lack of clinically relevant proarrhythmic risk related to QT prolongation further supporting the utility of C–QTc analysis. Based on this C–QTc modeling approach using early phase 1 study data as well as the totality of findings from other clinical and non‐clinical studies, fezolinetant was approved without conducting a dedicated TQT study.

## Author Contributions

Graeme L. Fraser and Steven Ramael made substantial contributions to the study design; Jace C. Nielsen and Masako Saito analyzed the study data; and Graeme L. Fraser, Steven Ramael, Jace C. Nielsen, Masako Saito, Xuegong Wang, Megumi Iwai, and Jiayin Huang interpreted the study data.

## Funding

This phase 1 study was conducted by Ogeda SA (formerly named Euroscreen SA) sponsorship. The additional analyses and publication were funded by Astellas Pharma Inc.

## Conflicts of Interest

All authors are employees of Astellas Pharma Global Development Inc. and Astellas Pharma Inc., and may hold stock or stock options.

## Supporting information



Supporting Information

## Data Availability

Details for how researchers may request access to anonymized participant level data, trial level data, and protocols from Astellas sponsored clinical trials can be found at https://www.clinicaltrials.astellas.com/transparency/
