# Supplementary material for: Concentration–QTc Modeling to Support Clinical Development of Fezolinetant
Source: Clin Pharmacol Drug Dev. 2025 Oct 19;15(1):e1613. doi: 10.1002/cpdd.1613 (PMC12811212; doi:10.1002/cpdd.1613)

**Concentration-QTc Modeling to Support Clinical Development of Fezolinetant**

Jace C Nielsen, PharmD^1^, Masako Saito, PhD^2^, Xuegong Wang, MD^1^, Megumi Iwai, PhD^2^, Graeme L Fraser, PhD^3*^, Steven Ramael, MD^3*^, Jiayin Huang, PhD^1*^

^1^ Astellas Pharma Global Development, Inc., IL, US

^2^ Astellas Pharma, Inc., Tokyo, Japan

^3^ Ogeda SA, Gosselies, Belgium

*Affiliation at the time of study

**Corresponding Author:**

Name: Jace Nielsen
Department: Early Development and Translational Science
Institution: Astellas Pharma Global Development, Inc.
Address: 2375 Waterview Drive, Northbrook, IL 60062
Email: jace.nielsen@astellas.com

**Abstract word count**: 169

**Manuscript word count**: 4171

**Tables and Figures**: 6 tables/figures

**References**: 18

**Supplemental Data**: 2 tables, 5 figures

# SUPPLEMENTAL DATA

## Supplemental Table S1 Parameter Estimates for the Parent Model

| Labels | Estimate | Standard Error | df | t value | p value |
| --- | --- | --- | --- | --- | --- |
| Intercept (msec) | -3.7828 | 2.4088 | 359 | -1.57 | 0.1172 |
| Treatment {Fezolinetant} (msec) | 2.2696 | 1.1097 | 407 | 2.05 | 0.0415 |
| Fezolinetant Slope (msec/(µg/mL)) | -0.5071 | 0.3116 | 74.3 | -1.63 | 0.1079 |
| Day {Day 7} (msec) | -2.4931 | 1.2715 | 427 | -1.96 | 0.0505 |
| Fezolinetant Slope * Day {Day 7} (msec/(µg/mL)) | 0.1449 | 0.3469 | 237 | 0.42 | 0.6766 |
| Deviation from Baseline (msec) | -0.3946 | 0.0520 | 174 | -7.59 | <.0001 |
| Nominal Time {1} (msec) | 0.9000 | 2.1347 | 389 | 0.42 | 0.6735 |
| Nominal Time {2} (msec) | 4.5197 | 2.1866 | 393 | 2.07 | 0.0394 |
| Nominal Time {4} (msec) | 5.5348 | 2.2242 | 396 | 2.49 | 0.0132 |
| Nominal Time {8} (msec) | -5.1046 | 2.1450 | 389 | -2.38 | 0.0178 |
| Nominal Time {24} (msec) | -0.4036 | 2.1216 | 386 | -0.19 | 0.8492 |
|  |  |  |  |  |  |
| Variance (Intercept) | 35.7109 | Not applicable | | | |
| Variance (Fezolinetant Slope) | 0.8752 |  |  |  |  |
| Covariance (Intercept, Fezolinetant Slope) | 1.0275 |  |  |  |  |
| Variance (Residual) | 33.6112 |  |  |  |  |

## Supplemental Table S2 Parameter Estimates for the Parent and Metabolite Model

| Labels | Estimate | Standard Error | df | t value | p value |
| --- | --- | --- | --- | --- | --- |
| Intercept (msec) | -1.9940 | 2.4783 | 256 | -0.80 | 0.4218 |
| Treatment {Fezolinetant} (msec) | 1.1068 | 1.2220 | 376 | 0.91 | 0.3656 |
| Fezolinetant Slope (msec/(µg/mL)) | -1.5319 | 0.3814 | 22.1 | -4.02 | 0.0006 |
| ES259564 Slope (msec/(µg/mL)) | 2.4566 | 0.7180 | 59.9 | 3.42 | 0.0011 |
| Day {Day 7} (msec) | -2.375 | 1.3351 | 359 | -1.78 | 0.0761 |
| Fezolinetant Slope * Day {Day 7} (msec/(µg/mL)) | 0.8064 | 0.4331 | 51.6 | 1.86 | 0.0683 |
| ES259564 Slope * Day {Day 7} (msec/(µg/mL)) | -1.6826 | 1.0513 | 197 | -1.60 | 0.1111 |
| Deviation from Baseline (msec) | -0.4590 | 0.05756 | 187 | -7.97 | <.0001 |
| Nominal Time {1} (msec) | 2.0382 | 2.1180 | 326 | 0.96 | 0.3366 |
| Nominal Time {2} (msec) | 5.5699 | 2.1718 | 330 | 2.56 | 0.0108 |
| Nominal Time {4} (msec) | 5.3632 | 2.2205 | 330 | 2.42 | 0.0163 |
| Nominal Time {8} (msec) | -6.5026 | 2.1574 | 328 | -3.01 | 0.0028 |
| Nominal Time {24} (msec) | -0.6063 | 2.1009 | 322 | -0.29 | 0.7731 |
|  |  |  |  |  |  |
| Variance (Intercept) | 39.3215 | Not applicable | | | |
| Variance (Fezolinetant Slope) | 0.03077 |  |  |  |  |
| Variance (ES259564 Slope) | 2.1821 |  |  |  |  |
| Variance (Residual) | 32.3441 |  |  |  |  |

Supplemental Figure S1 Mean Change from Baseline Heart Rate Versus Time

##
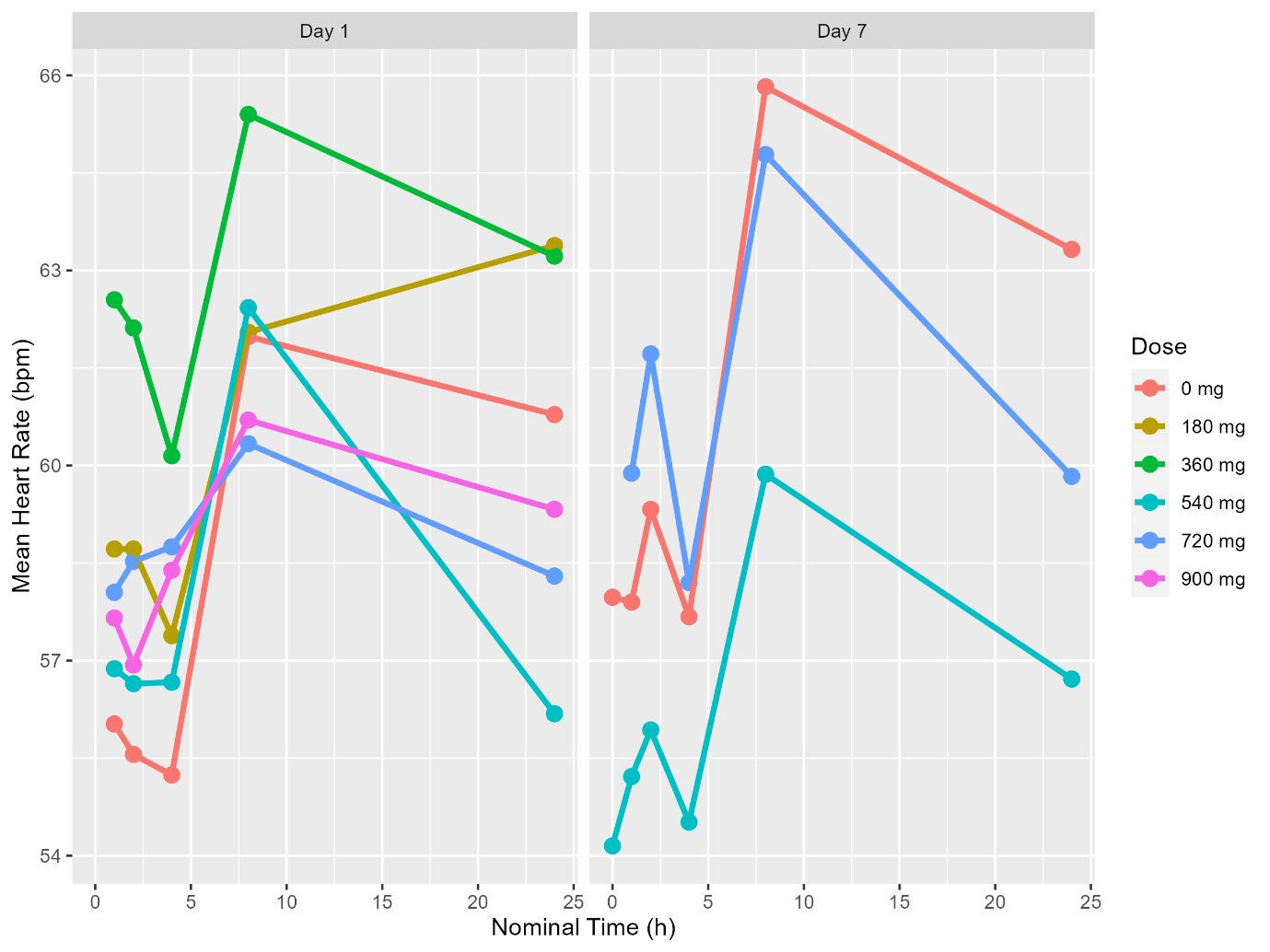


## Supplemental Figure S2 Evaluation of the Heart Rate Corrected QT Interval


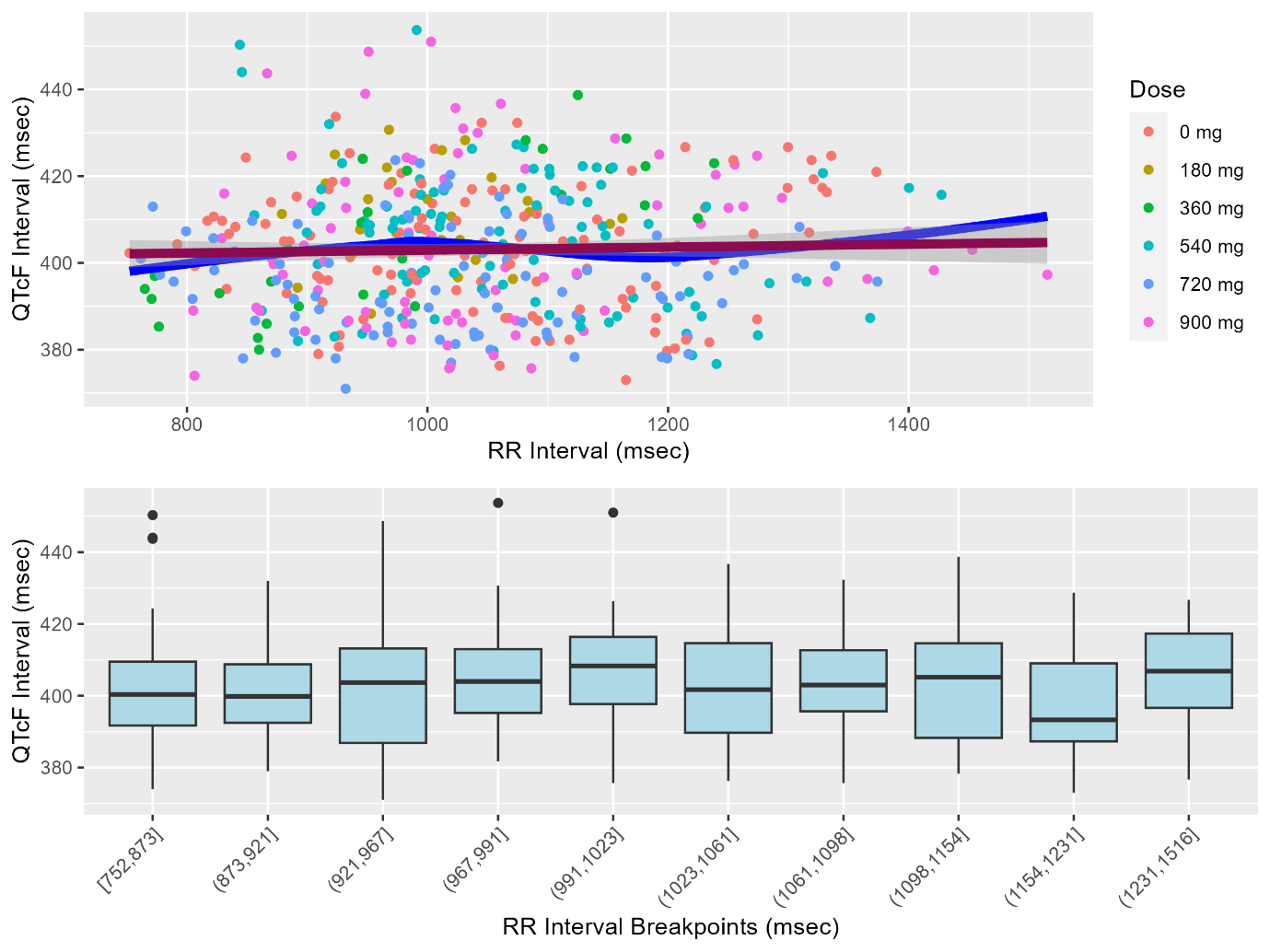


QTcF, Fridericia-corrected QT.

Top plot is a scatter plot of QTcF intervals vs RR intervals. Dark red line is linear regression line. Blue line is LOESS smooth. Light grey band is 95 percent CI for mean prediction. Bottom plot is a box and whisker plot of the top plot categorized into RR interval deciles.

## Supplemental Figure S3 Evaluation of Hysteresis


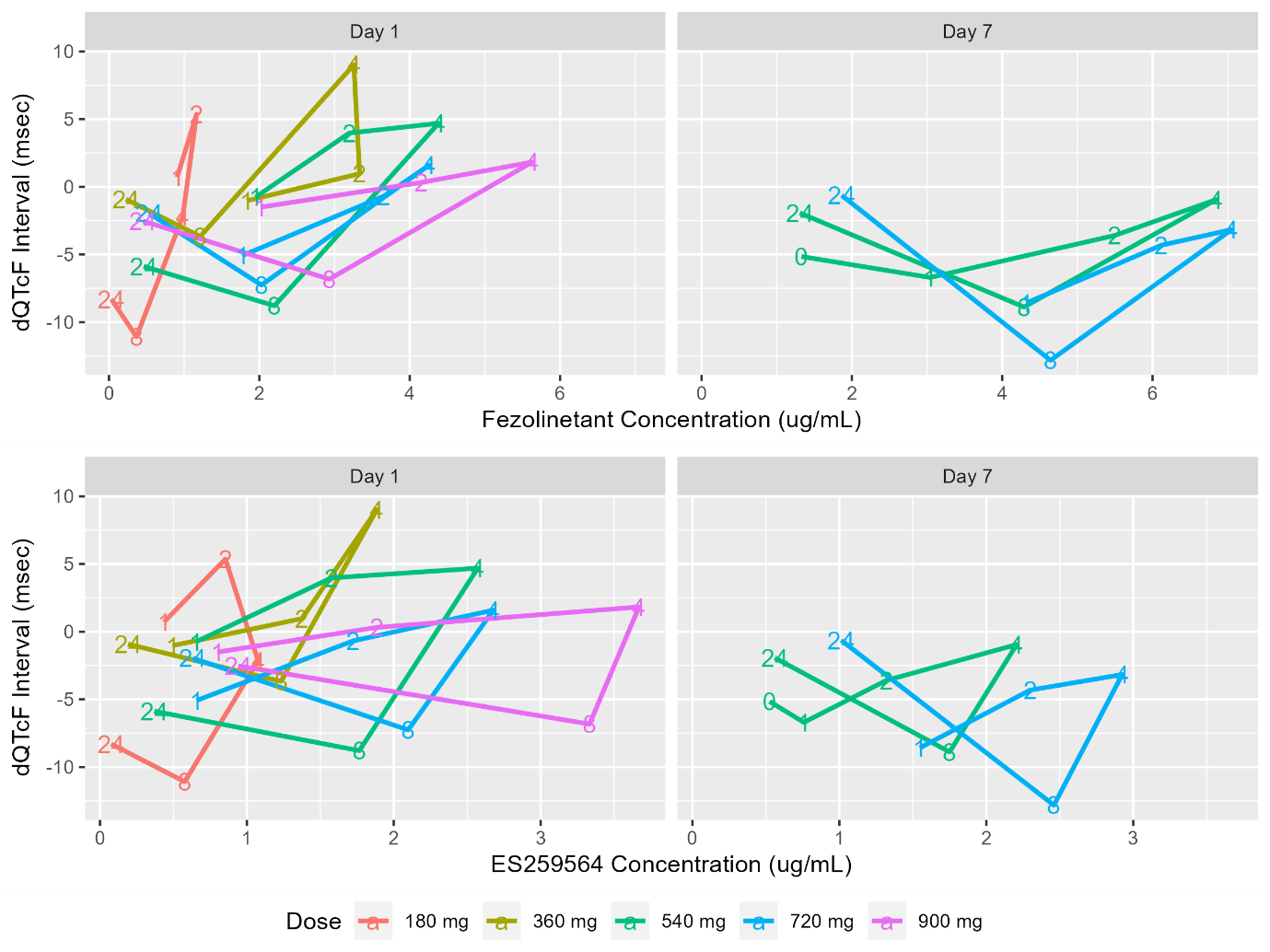


## Supplemental Figure S4 Diagnostic Plots for the Parent Model


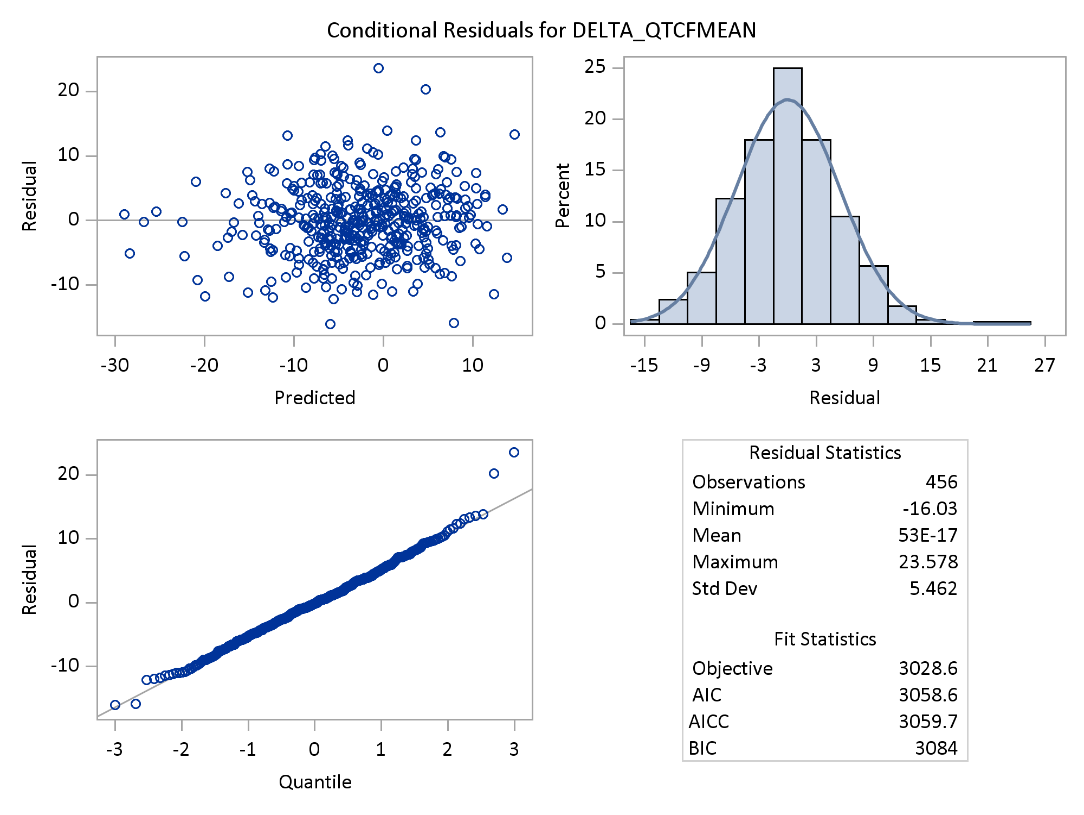


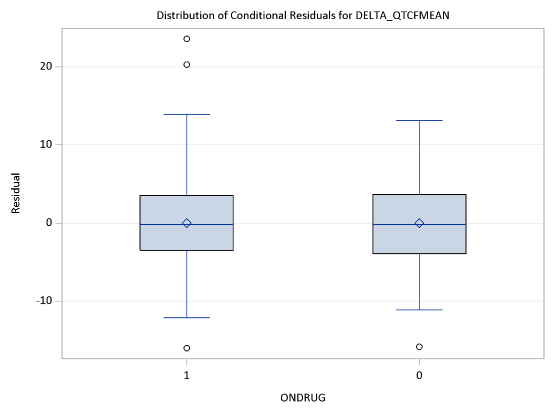

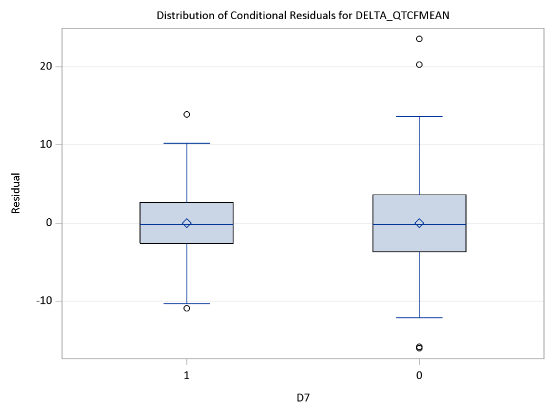


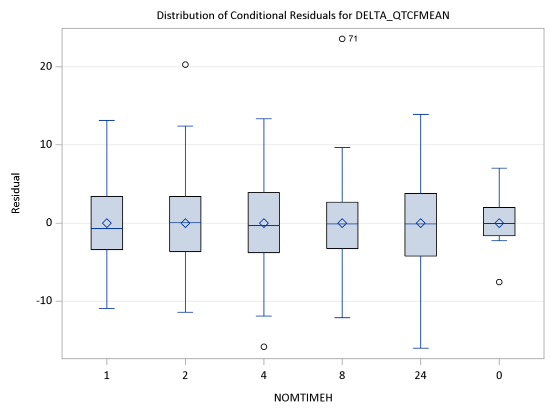

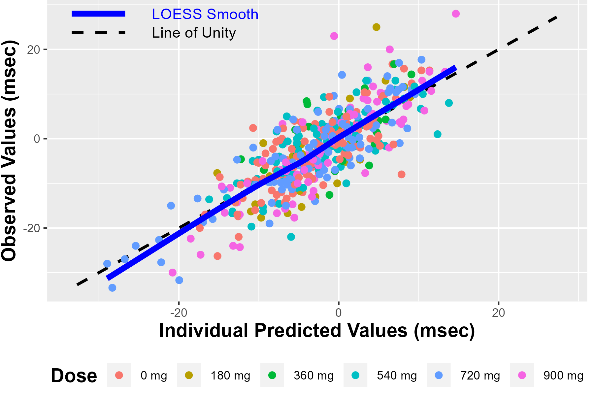


## Supplemental Figure S5 Diagnostic Plots for the Parent and Metabolite Model


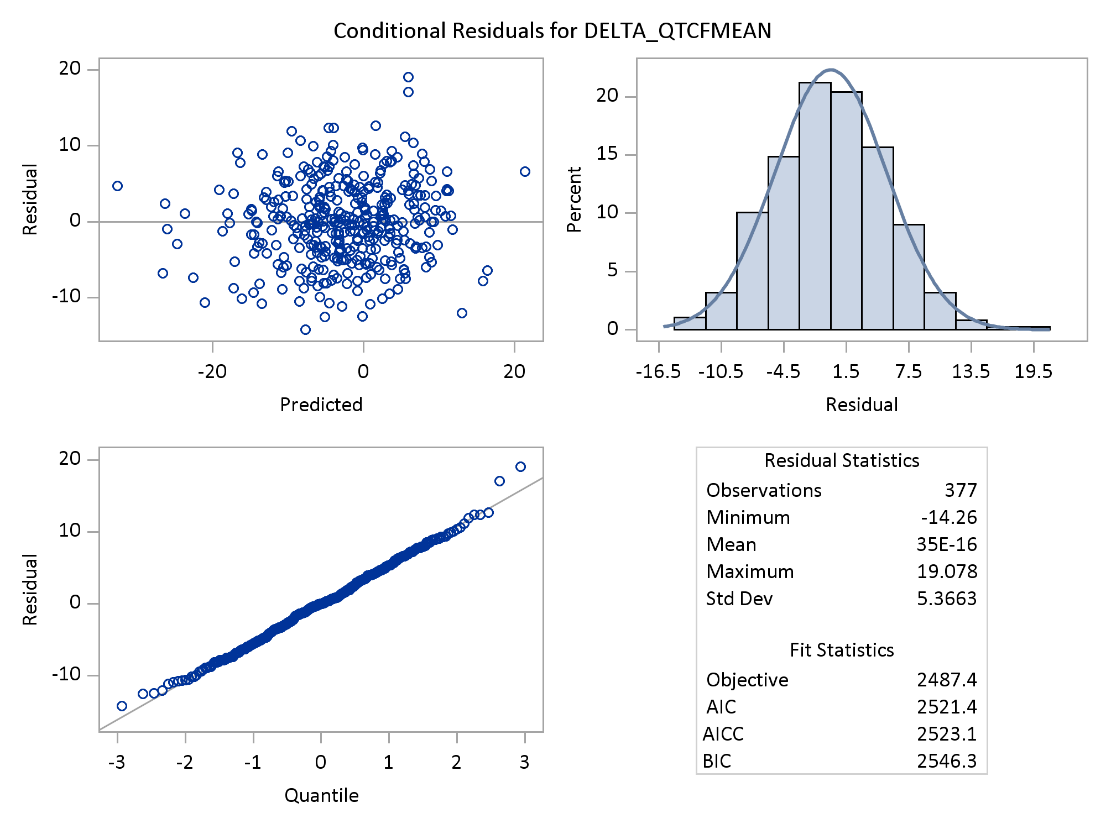


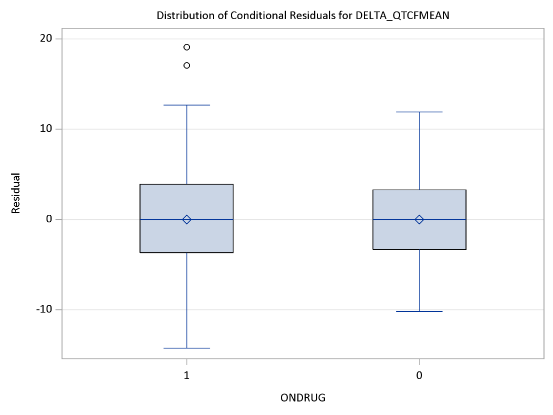

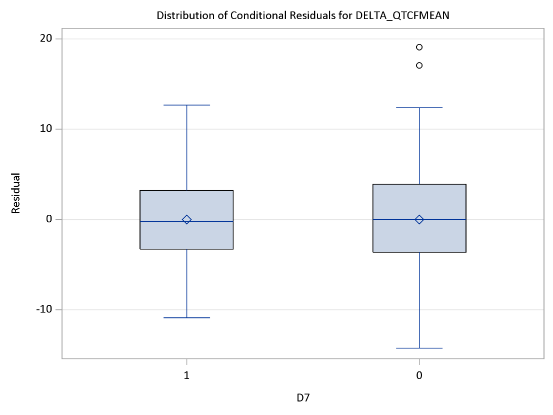


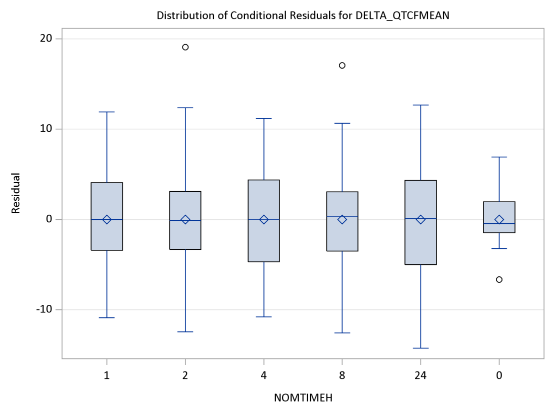

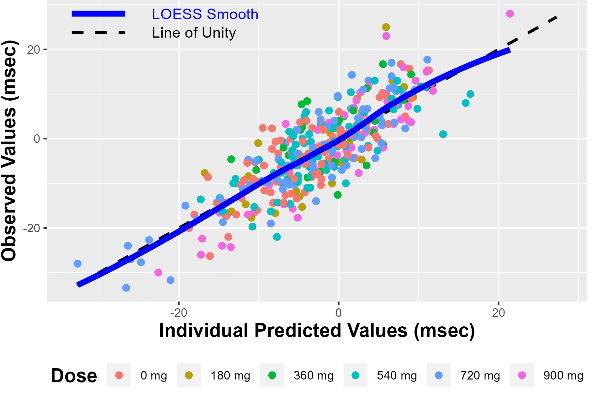

Supplement: Supplementary file 1 — Supporting Information [file CPDD-15-0-s001.docx]
